# Is pre-operative imaging essential prior to ureteric stone surgery?

**DOI:** 10.1308/003588412X13373405385458

**Published:** 2012-09

**Authors:** FR Youssef, BA Wilkinson, KJ Hastie, J Hall

**Affiliations:** Sheffield Teaching Hospitals NHS Foundation Trust,UK

**Keywords:** Urolithiasis, Ureteroscopy, Imaging

## Abstract

**INTRODUCTION:**

The aim of this study was to identify patients not requiring ureteric stone surgery based on pre-operative imaging (within 24 hours) prior to embarking on semirigid ureteroscopy (R-URS) for urolithiasis.

**METHODS:**

The imaging of all consecutive patients on whom R-URS for urolithiasis was performed over a 12-month period was reviewed. All patients had undergone a plain x-ray of the kidney, ureters and bladder (KUB), abdominal non-contrast computed tomography (NCCT-KUB) or both on the day of surgery.

**RESULTS:**

A total of 96 patients were identified for the study. Stone sizes ranged from 3mm to 20mm. Thirteen patients (14%) were cancelled as no stone(s) were identified on pre-operative imaging. Of the patients cancelled, 8 (62%) required NCCT-KUB to confirm spontaneous stone passage.

**CONCLUSIONS:**

One in seven patients were stone free on the day of surgery. This negates the need for unnecessary anaesthetic and instrumentation of the urinary tract, with the associated morbidity. Up-to-date imaging prior to embarking on elective ureteric stone surgery is highly recommended.

Urolithiasis has a prevalence of 1–2% in the general population,^1^ with an annual incidence of 15–20 per 10,000. Males are three times more likely to form stones than females. The lifetime risk of developing a renal tract calculus is 12% in white males.

Ureteric stone disease often presents with symptoms of ureteric colic (characteristically), loin to groin pain, nausea and sometimes vomiting. A history of previous stone disease should also be ascertained. Diagnostic imaging should be obtained to support the clinical diagnosis. Helical non-contrast computed tomography of the kidney, ureters and bladder (NCCT-KUB) has become the gold standard for the radiological diagnosis of urolithiasis.^2^

Ureteric calculi can pass spontaneously. The size, site and shape are determinant factors in the rate of spontaneous stone passage. For smaller stones, where the spontaneous passage rate is high (80% of calculi <4mm), a conservative approach is generally the initial management with intervention for those that fail to pass.^3^ For patients with larger stones, where the spontaneous passage rate is much lower, the initial treatment plan is interventional. Among patients for whom active intervention has been planned, there are still some who pass their stone spontaneously. Spontaneous stone passage has been seen in 25–60% of patients with stone diameters between 5mm and 10mm.^4^ Spontaneous passage has also been seen in 48% of patients with proximal ureteric stones, 60% of those with mid-ureteric stones and 75% of those with distal ureteric stones.

The need for pre-operative imaging to confirm the ongoing presence of calculi was the subject of some debate at the British Association of Urological Surgeons Section of Endourology meeting in Norwich in 2009. An informal discussion of the floor during a live surgical procedure confirmed that some units have abandoned pre-operative imaging for ureteric stone cases.

Semirigid ureteroscopy (R-URS) is not without a small but significant risk of morbidity such as sepsis (1%) and ureteric stricture (1–2%).^5^ This risk doubles if an iatrogenic ureteric perforation occurs and, in rare cases, it can lead to mortality.

The aim of this study was to identify the number of patients not requiring R-URS stone surgery following pre-operative imaging as well as the imaging modalities used to determine when surgery is no longer required.

## Methods

All patients listed for R-URS for ureteric calculi over a 12-month period from June 2008 to June 2009 were identified from the theatre Operating Room Management Information System (ORMIS) register. Diagnostic imaging at presentation was reviewed in all patients to determine the size, site and number of ureteric calculi, as was any imaging in the interim period prior to surgery. All patients admitted for elective R-URS underwent either a plain x-ray of the kidney, ureters and bladder (KUB) or NCCT-KUB or, occasionally, both on the day of proposed surgery. This was established practice in our institution at the time of the study. KUB was performed for radio-opaque calculi and NCCT-KUB for radiolucent calculi. All imaging was reviewed by the operating surgeon prior to theatre. All patients cancelled within 24 hours of surgery as a result of no stones on their imaging were identified from the ORMIS register.

## Results

In total, 96 patients were listed for R-URS for ureteric calculi. Stone sizes ranged from 3mm to 20mm, with a median size of 7mm. In 28 cases (29%), stones were radiolucent on plain KUB but were seen on NCCT-KUB at the time of diagnosis, thus necessitating NCCT-KUB on the day of admission. Pre-operatively, 57 patients (59%) had plain KUB only, 29 (30%) had NCCT-KUB only and 10 (10%) had both.

**Figure 1:**
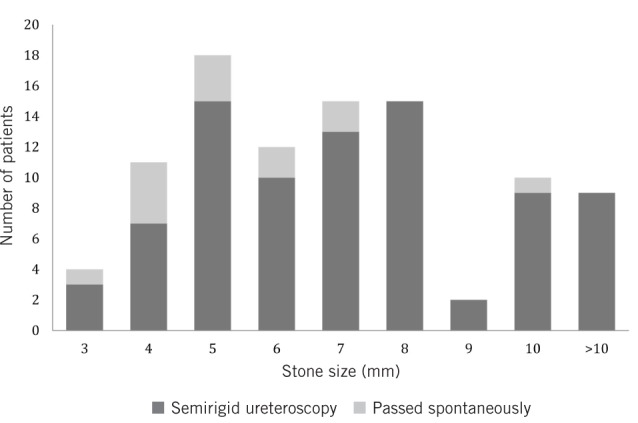
Stone sizes in patients who underwent semirigid ureteroscopy and those in whom spontaneous stone passage occurred

Overall, 13 patients (14%) were cancelled within 24 hours prior to elective surgery due to no stones being identified on pre-operative imaging. Stone sizes in the spontaneous passage group ranged from 3mm to 10mm (Fig 1), with a median size of 5mm. Of the 13 patients cancelled, 8 (62%) required NCCT-KUB to confirm no stone was present (Table 1).

**Table 1:** Distribution of radio-opaque and radiolucent stones and the pre-operative imaging modality performed

	Radio-opaque stones	Radiolucent stones	Total
All stones	68 (71%)	28 (29%)	96 (100%)
KUB	57	0	57 (59%)
NCCT-KUB	7	22	29 (30%)
Both	4	6	10 (10%)
Passed spontaneously	8	5	13 (14%)
KUB	5	0	5
NCCT-KUB	1	2	3
Both	2	3	5

KUB = kidney, ureters and bladder x-ray; NCCT-KUB = kidney, ureters and bladder non-contrast computed tomography

## Discussion

A significant proportion of patients were found to have passed their stone(s) spontaneously as discovered on pre-operative imaging prior to elective surgery. This can occur in a wide variety of stone sizes and location.^4^ Our study included a patient who spontaneously passed a stone of 10mm in size. In our experience, impacted stones can be smaller yet we know larger stones are able to pass spontaneously in certain individuals. Pre-operative imaging is worthwhile in all patients to negate unnecessary urinary tract instrumentation. NCCT-KUB was required to identify almost two-thirds of those patients cancelled. This avoided a negative R-URS for 1 in 7 patients, consequently preventing them undergoing unnecessary hospital admission, anaesthesia and urinary tract instrumentation. This would expose the patient to the associated risks and morbidity. The overall complication rate of R-URS has been reported as high as 6%, with a 1% risk of major complications.^6^

In our institution, plain KUB and NCCT-KUB are performed under standard protocols. The radiation exposure for a single KUB is 0.7mSv and for NCCT-KUB it is 5mSv.^7^ The mean dose of radiation exposure per patient was 7.04mSv (95% confidence interval [CI]: 6.44–7.64mSv) prior to any pre-operative imaging being performed. Patients routinely underwent NCCT-KUB and KUB at acute presentation of ureteric colic. Twenty-four patients underwent interim extracorporeal shock wave lithotripsy (ESWL). This was performed on radio-opaque calculi with a KUB prior to ESWL. Eighteen patients required multiple sessions of ESWL.

The cumulative mean radiation exposure following pre-operative imaging was 9.43mSv (95% CI: 8.69–10.20mSv). An online radiation exposure calculator was used to assess the additional risk of cancer based on age, sex and imaging modalities along with the radiation dose for each modality.^8^ The mean increase in lifetime risk of developing cancer was 0.01% (95% CI: 0.009–0.015%). This is in addition to an individual’s baseline risk if pre-operative imaging alone is performed prior to R-URS.

A negative R-URS carries with it a small but significant risk of morbidity with complications being reported in up to 6% of cases,^6^ such as sepsis (1%) and ureteric stricture (1–2%). This risk doubles if an iatrogenic ureteric perforation occurs and in rare cases it can lead to mortality.^5^ When balancing the risks of surgery combined with anaesthesia with the risks of pre-operative imaging, we feel that the risks of surgery and anaesthesia outweigh those of increased radiation exposure due to pre-operative imaging alone.

In total, 17 patients underwent interim percutaneous nephrostomy (PCN) tube insertion and 4 underwent ureteric stent insertion under fluoroscopy guidance between acute presentation and planned R-URS. Of those 39 patients who underwent pre-operative NCCT-KUB, 10 had PCN tubes in situ and 2 had ureteric stents in situ. Spontaneous stone passage occurred in one patient with a PCN in situ and, consequently, R-URS was not performed. The two patients with ureteric stents underwent R-URS as their stones remained present on pre-operative imaging.

Of the 83 patients who proceeded to R-URS, 5 (6%) underwent unplanned NCCT-KUB on the day following KUB to confirm the presence of their calculi. Sensitivity of plain KUB has been reported as low as 54%.^9^ If there was any diagnostic uncertainty, NCCT-KUB was requested after discussion with a consultant uroradiologist on the day of surgery to confirm the presence of a calculus prior to proceeding to elective R-URS.

Patients were treated on the basis of their pre-operative imaging on the day of surgery. If this did not demonstrate any evidence of calculi, patients did not undergo R-URS, irrespective of symptoms, to avoid the risk associated with urinary tract instrumentation in the absence of identifiable calculi. In cases where no stones were found on R-URS, without adequate pre-operative imaging, some may have proceeded to flexible ureteroscopy (F-URS) for fear of the stone having refluxed up into the kidney, involving increased instrumentation and anaesthetic time.

This study was performed in a tertiary referral centre. Some cases were complex due to hostile anatomy such as severe scoliosis and congenital dysplasia of the hip. If any uncertainty arose on pre-operative KUB, further NCCT-KUB was requested if necessary, often after consultation with a uroradiologist. These cases had pre-operative NCCT-KUB to ensure clear delineation of their anatomy as well as confirming the presence of a stone.

Operating lists are coordinated accordingly to allow time for patients to undergo planned pre-operative imaging. Plain KUB can be performed promptly before operating lists are due to commence. Those patients requiring NCCT-KUB are placed further down the lists to allow time for imaging to be performed and reviewed. If additional imaging is required, prompt communication and a good working relationship with the radiology department ensures there is no delay in the progression of the operating list. Reordering of the lists can occur without undue disruption.

Other benefits were found on performing thorough pre-operative imaging:

1. A patient known to have right ureteric and left renal calculi was admitted for right R-URS and stone fragmentation. However, pre-operative imaging did not demonstrate the right ureteric calculus. The patient instead proceeded to have F-URS and stone fragmentation for the known left renal calculus.
2. Pre-operative NCCT-KUB for a radiolucent ureteric calculus had shown this had migrated proximally up to the pelvic ureteric junction. F-URS was therefore required.
3. A patient with two radiolucent ureteric calculi at diagnosis demonstrated a single calculus only on pre-operative NCCT-KUB imaging.
4. A patient with a single ureteric calculus at diagnosis demonstrated three ureteric calculi on pre-operative NCCT-KUB.

In the above cases, the treatment plan was altered and different techniques were used to treat their stones. Prior notice could thus be given to theatre staff in readiness of scopes and equipment as well as informing the anaesthetist. This ensures efficient use of theatre time and running of theatre lists. Pre-operative imaging also confirms stone number. In this study, the stone number for two patients changed between initial presentation and elective surgery. This avoided pursuing calculi that had passed spontaneously prior to surgery as well as the premature completion of surgery due to the presence of calculi unknown to the operating surgeon, which would have remained untreated.

## Conclusions

Pre-operative imaging is essential in the surgical management of ureteric stone disease. One in seven patients (14%) was found not to have stones on pre-operative imaging. Of those patients cancelled, 8 (62%) required NCCT-KUB to prove stone passage had occurred within 24 hours prior to surgery, confirming the value of NCCT-KUB in the pre-operative assessment. We feel this is safer than relying on negative KUB. This avoids unnecessary anaesthesia and instrumentation of the urinary tract, with the associated potential morbidity. Up-to-date imaging prior to embarking on elective ureteric stone surgery is highly recommended.
